# Expression, Mutation, and Amplification Status of EGFR and Its Correlation with Five miRNAs in Salivary Gland Tumours

**DOI:** 10.1155/2017/9150402

**Published:** 2017-03-09

**Authors:** Emanuela Boštjančič, Nina Hauptman, Aleš Grošelj, Damjan Glavač, Metka Volavšek

**Affiliations:** ^1^Department of Molecular Genetics, Institute of Pathology, Faculty of Medicine, University of Ljubljana, Zaloška Cesta 4, 1000 Ljubljana, Slovenia; ^2^Department of Otorhinolaryngology and Cervicofacial Surgery, University Medical Centre Ljubljana, Zaloška Cesta 2, 1000 Ljubljana, Slovenia; ^3^Institute of Pathology, Faculty of Medicine, University of Ljubljana, Korytkova 2, 1000 Ljubljana, Slovenia

## Abstract

Malignant salivary gland tumours are rare histologically and clinically heterogeneous group of tumours, missing prognostic factors and therapeutic targets. MicroRNAs (miRNAs), small noncoding RNAs, and posttranscriptional regulators of mRNA are poorly described in different subtypes of salivary gland tumours. Epidermal growth factor receptor (EGFR), an important therapeutic target and target of certain miRNAs (i.e.,* miR-133b*), shows variable degrees of expression in salivary gland tumours. Our study included 70 parotid gland tumours of different histological subtypes. Expression, mutations, and copy number variations (CNVs) of EGFR were determined using immunohistochemistry, single-stranded conformation polymorphism, quantitative polymerase chain reaction (qPCR), and fluorescence in situ hybridization. Expression of* miR-99b*,* miR-133b*,* miR-140*,* miR-140-3p*, and* let-7a* was analysed using qPCR. Expression of EGFR was observed in 37% of tumours with low and 40% of tumours with high malignant potential. There were no mutations, with the majority of samples showing polysomy of chromosome 7. Based on histological subtypes, we found differential expression of all five miRNAs. We confirmed association of reactivity of EGFR,* miR-133b*,* miR-140*,* miR-140-3p*, and* let-7a* with CNV of EGFR and a positive association between* miR-133b*/*let-7a* and reactivity of EGFR. Age and need for postoperative radiotherapy were characterized as significant in multivariate survival analysis.

## 1. Introduction

Malignant salivary gland tumours are rare tumours with an annual incidence of 0.4–2.6/100.000 and represent ~6% of all head and neck tumours [1]. This is a very heterogeneous group of tumours in terms of histological subtypes and diverse clinical behaviour and prognoses [2]. The primary treatment option in localized disease is surgery, with decision on postoperative radiotherapy depending upon histological type and tumour stage. Since in some patients relapsing or metastatic disease develops, there is a need for new therapeutic strategies [3].

Abnormalities within human genome that might contribute to a wide range of cancer types may include small- and large-scale genomic alterations. Oncogenes and tumour-suppressors, including protein-coding genes [4] as well as noncoding RNAs [5], can be affected by numerous genetic alterations. These alterations include chromosomal translocations and rearrangements, copy number variations, mutations within genes, expansion of nucleotides, and single-nucleotide polymorphisms. Recently, chromosome rearrangements in diagnostic pathology of salivary gland malignancies have been summarized [6]. However, there are still unexplained cases, missing prognostic factors, and therapeutic targets that have to be defined in salivary gland tumour pathology.

Epidermal growth factor receptor (EGFR) is a trans-membrane tyrosine kinase, an oncogene that is often continuously activated and consequently activates a series of intracellular signalling cascades. Activation of cascades often affects gene transcription, resulting in angiogenesis, cell differentiation, proliferation, and tumour progression of many cancers. Overexpression of* EGFR* is usually due to amplification of gene or activating mutations within the gene. Therefore, in non-small cell lung cancer and colorectal cancer it is important also as a therapeutic target, where in case of mutation either tyrosine kinase inhibitors or monoclonal antibodies against EGFR are used in routine practice [2]. Several experiments performed were describing expression of EGFR in salivary gland tumours, however, with variable degrees of expression being reported so far, from 17% to 100% of cases, depending on histological subtypes [1, 3].

Other important players in tumorigenesis are miRNAs, small noncoding RNAs. In association with proteins miRNAs form miRNA-ribonucleoprotein complex, bind to 3-UTR of target mRNA, and thus sufficiently block translation. miRNA may be ubiquitously expressed or might possess cell- or tissue-specific expression pattern. miRNAs thus function in numerous biological processes from development to adult life, including proliferation, differentiation, division of stem cells, apoptosis, and epithelial-to-mesenchymal transition (EMT) [7, 8]. Studying cancer phenotype, it was also shown that approximately ~50% of the miRNA genes are encoded within sites that are prone to cancer-associated rearrangements [9]. Genomic abnormalities often cause abnormal miRNA expression profiles. Numerous expressions profiling, using microarrays and next gene sequencing approaches of miRNA analysis, suggests that miRNAs are associated with various types of cancer [10], including salivary gland tumours. One of the first studies described expression of Dicer, a miRNA maturation enzyme, in mucoepidermoid carcinoma (MEC), a subtype of a malignant salivary gland tumour. Abnormal immunoexpression of Dicer suggested a role for miRNAs in tumour progression of MEC [11]. Another study described alterations in miRNA expression in pleomorphic adenomas (PAs), the most frequent benign salivary gland tumours.* miR-140* and* miR-99b* were described among mostly upregulated miRNAs, and* let-7a* was mostly downregulated [12].* miR-133b*, among other miRNAs, target EGFR in different cancer types, especially non-small cell lung cancer [13, 14].

Taken together, aims of this study were (i) to investigate expression of* EGFR* in different subtypes of salivary gland tumours, (ii) to define whether overexpression is correlated with the copy number variation of* EGFR* (amplification of* EGFR *or polysomy of chromosome 7) or mutation within* EGFR* gene, (iii) to analyse whether expression of* miR-99b*,* miR-140,* and* let-7a* is different between different salivary gland carcinoma subtypes, and finally (iv) to test whether expression of* miR-133b* is in correlation with the expression of their target, EGFR, also in salivary gland carcinoma.

## 2. Materials and Methods

### 2.1. Patients and Tissue Samples

The study comprised 70 patients with carcinomas of the parotid gland, diagnosed at the Institute of Pathology, Faculty of Medicine at the University of Ljubljana, between 1995 and 2008. At diagnosis, patients were staged according to the 6th edition of UICC/TNM system [15]. Tumour surgery was performed at the Department for Otorhinolaryngology and Cervicofacial Surgery at University Clinical Centre Ljubljana.

Haematoxylin-eosin (HE) slides from formalin-fixed paraffin-embedded (FFPE) tumours were available for all cases and reviewed by pathologist, who chose representative tissue blocks for further analysis.

For all samples histological subtypes were determined. Additionally, eleven normal salivary glands were included as tissue controls. All the tumour samples contained more than 50% of tumour tissue, with majority of samples between 70 and 80%.

The study was approved by Republic of Slovenia National Medical Ethics Committee (number 77/04/13).

### 2.2. Immunohistochemistry

FFPE tissue samples were cut at 4 *µ*m for immunohistochemistry (IHC). All reagents were from Ventana Medical Systems Inc., Tucson, USA, except otherwise indicated. We used commercially available antibodies against EGFR (CONFIRM™ anti-EGFR, clone 3C6). Deparaffinization, antigen retrieval (including 8 minutes of Protease 1), and staining with antibodies were performed in an automatic immunostainer (Benchmark XT) in combination with treating sections with secondary antibody and colour development with horseradish peroxidase (ultraVIEW DAB Detection Kit). The sections were then counterstained with haematoxylin. Tissue sections of normal salivary glands served as positive and sections treated without primary antibodies as negative controls.

The immunostaining of EGFR was semiquantitatively evaluated based on intensity of membrane reactivity with a threshold of 10% immune-positive cells [1]: 0, negative (no reactivity or reactivity in <10% of cells); 1+, weak reactivity in >10% of cells; 2+, moderate reactivity in >10% cells; and 3+, strong reactivity in >10% cells. Tumours with 3+ staining were considered as positive. Immunostaining pattern was also documented in normal salivary gland tissues as controls. All tissue samples were stained and analysed in duplicate.

### 2.3. DNA Isolation

Tissue samples were cut at 10 *μ*m from FFPE tissue blocks and for the isolation procedure, six to eight 10 *μ*m sections were used. Total DNA isolation was performed using QIAamp DNA FFPE Tissue Kit (Qiagen) according to the manufacturer's protocol. The DNA was eluted in 60 *μ*l of elution buffer. The yield was measured fluorescently using Quant-It (Invitrogen) according to manufacturer instruction and Rotor Gene Q (Qiagen).

### 2.4. Copy Number Variation (CNV)

The copy numbers for EGFR were determined using commercially available and predesigned TaqMan Copy Number Assays according to the manufacturer's instructions (Applied Biosystems). All reagents were from Applied Biosystems except otherwise indicated. The primer ID used for EGFR was as follows: EGFR1, Hs00997424_cn [16]. The RNaseP and TERT locus were used as the Copy Number Reference Assays. Human Genomic Control DNA (10 ng/*µ*l) was used as a normal control (calibrator sample). Prior to CNV PCR reaction, efficiency was tested for all three genes as well as for Human Genomic Control DNA and pooled FFPE samples. Real-time genomic PCR was performed as duplex PCR in a total volume of 10 *µ*l in each well, containing 5 *µ*l of TaqMan Universal PCR master mix, 10 ng of genomic DNA, and 0.5 *µ*l of each pair of primers (20x TaqMan Copy Number Assay for EGFR and 20x TaqMan Copy Number Assay for either TERT or RNaseP). Each reaction was performed in duplicate using Rotor Gene Q (Qiagen). The PCR conditions were 95°C for 10 min and 40 cycles of 95°C for 15 s and 60°C for 1 min. Data were analysed according to Applied Biosystems recommendation. Since efficiencies were comparable, each replicate was normalized to RNAaseP or TERT to obtain a ΔCt (Ct_TERT/RNaseP_ − Ct_EGFR_) and then averaged for each sample. All samples were then normalized to a calibrator sample to obtain ΔΔCt. Copy number was calculated as follows: 2xRQ, where RQ stands for relative quantity (2^−ΔΔCt^).

### 2.5. Fluorescent In Situ Hybridization (FISH)

For FISH analysis 20 samples were randomly selected to compare to results from Copy number variation. 1-2 *µ*m sections were mounted on charged slides (Thermo Scientific Super Frost Glass). HE slides were used for reference histology. For FISH analysis reagents from Vysis, Abbot Molecular was used, except otherwise indicated. After deparaffinization using xylene (Merck), slides were incubated 20 min in 0.2 M HCl (Merck), following washing in water and 2xSSC and incubation in NaSCN for 30 min at 80°C. Prior to protease digestion for 30 min at 37°C (Paraffin Pretreatment Kit II) washing in water and 2xSSC was repeated. After protease digestion and washing in 2xSSC slides were dried for 5 min at 57°C and incubated for 10 min in 10% buffered formalin. Following washing in 2xSCC and water, slides were incubated in fixative methanol acetic acid (4 : 1) and washed in 70% and 100% ethanol. Fluorescence in situ hybridization (FISH) was performed with the use of directly labelled ZytoLight SPEC EGFR/CEN7 dual colour probes (ZytoVision) according to manufacturer instruction. Codenaturation and hybridization were performed in ThermoBrite using following conditions: 73°C 5 min and 37°C overnight. After probe hybridization, slides were washed in 2xSSC/0.1NP40 for 2 min, in 2xSSC/0.3NP40 for 2 minutes at 73°C, and for 1 min in 2xSSC/0.1NP40. Nuclei were counterstained with antifading 4′,6-diamidino-2-phenylindole (DAPI) and were analysed using the Eclipse E600 microscope (Nikon). Hybridization signals of 30–100 nonoverlapped nuclei were manually counted on single cell basis.

For EGFR, samples were grouped as normal disomy, ≤2 centromere signals in ≥40% of cells; low polysomy/trisomy, ≥3 centromere signals in ≥40% of cells, excluding cases with high polysomy or gene amplification; high polysomy, ≥4 centromere signals in ≥40% of cells, excluding cases with gene amplification; and gene amplification, ratio of gene/chromosome ≥2 or clusters of probes (>10 copies per tumour cell) in ≥40% of cells.

### 2.6. Polymerase Chain Reaction (PCR)

Five pairs of nucleotide primers were selected in order to obtain molecular analysis of exons 18, 19, 20, and 21 of EGFR gene (Table 1). The primers were prepared with IDT tools and gene runner program and made at Eurofins and Qiagen Operon.

### 2.7. Single-Stranded Conformation Polymorphism (SSCP)

It was performed on the thin (0.4 mm) 6% or 8% nondenaturating polyacrylamide gel with 2.6% cross-linking. Prior to conformation analysis, six microliters of PCR products were mixed with 10 microliters of SSCP loading dye (95% formamide, 20 mM EDTA, 0.05% bromophenol blue, 0.05% xylene cyanol, and 20 mM NaOH) and heated to 90°C for 2 min. After loading on the polyacrylamide gel, the samples were electrophoresed in a 1xTBE (TrisBorateEDTA) buffer in a cold room (4°C) for 4-5 hours at 50 W and silver stained.

### 2.8. Sequence Analysis

ABI Prism 310 Genetic Analyzer (Applied Biosystems) was used to analyse sequences. The reaction was performed in a volume of 10 microliters using BigDye Terminator v1.1 Cycle Sequencing Kit (Applied Biosystems) containing dideoxynucleotide triphosphate. After reaction was completed the products were cleaned with sodium acetate method.

### 2.9. RNA Isolation from FFPE Tissue Samples

Tissue samples were cut at 10 *μ*m from FFPE tissue blocks and for the isolation procedure, six to eight 10 *μ*m sections were used. Total RNA isolation was performed using miRNA easy FFPE Kit (Qiagen) according to the manufacturer's protocol. The RNA was eluted in 30 *μ*l of nuclease-free water. The yield was measured spectrophotometrically using NanoDrop-1000 (Thermo Scientific, USA) and the quality was evaluated on Bioanalyzer 2100 (Agilent Technologies, USA).

### 2.10. Quantitative Real-Time PCR (qPCR)

All the reagents were from Qiagen, except where otherwise indicated. Quantitative PCR (qPCR) was carried out using the Rotor Gene Q Real-Time PCR System.

Prior to qPCR analysis, two pools of RNA samples were created from FFPE tissue samples (salivary gland tumour and normal salivary gland tissue). After reverse transcription of pooled RNA and reference RNA, the cDNA was diluted in five steps and the probes were tested for qPCR efficiency. All the qPCR efficiency reactions were performed in triplicate.

For reverse transcription the miScript reverse transcription kit was used. Briefly, a 10 *μ*l reaction master mix was performed, containing 50 ng of total RNA, 2 *μ*l 5x miScript HiFlex RT buffer, 1 *μ*l miScript reverse transcriptase mix, 1 *μ*l miScript nucleic mix, and 10 units (0.33 *μ*l) RNase inhibitor. After incubation for 60 min at 37°C and 5 min at 95°C, the cDNA was diluted 100-fold, and 3 *μ*l was used for each qPCR reaction. Ten *μ*l PCR master mix contained 5 *μ*l 2x QuantiTect SYBR Green PCR Master Mix, 1 *μ*l miScript universal primer, and 1 *μ*l 10x miScript Primer Assay. As the reference genes, RNU5A, RNU6B, and SNORD25 were used based on pretesting results. All the qPCR reactions were performed in duplicate. The signal was collected at the endpoint of every cycle. Following amplification, melting curves analysis of PCR products was performed to verify their specificity and identity. Melting curves were acquired on the SYBR channel using a ramping rate of 0.7°C/60 s for 60–95°C. Tested microRNAs were* miR-99b*,* miR-133b*,* miR-140*,* miR-140-3p*, and* let-7a*. To present a relative gene expression the 2^−ΔΔCt^ method was used, in which the fold changes of the tested groups were calculated relative to the calibrator group [17, 18].

### 2.11. Statistical Analysis

For expression analysis of independent group of samples (i.e., salivary gland tumour, normal salivary gland), ΔCt was used for Mann–Whitney test. The data were presented as a fold change in a graph with error bars representing the calculated fold change error using the SD of the Ct triplicates.

The expression level of EGFR was determined by IHC, which was scored by multiplying the intensity (range 0–3) with the percentage of positive cells (range 0%–100%). Scored expression levels of EGFR, expression of miRNA, and copy number of EGFR were tested for correlation using polycor package in R (Drasgow, 1986). Pearson's correlation coefficient and *p* values were calculated. Polyserial correlation between ordinal variables (stage, tumour size, and presence/absence of metastasis) and the expression of EGFR, miRNAs, and CNV of EGFR were calculated using the polyserial function in the polycor library in R.

Overall survival analysis was performed in software R, with the latest data of patients obtained in year 2015. Survival was estimated using Cox proportional hazards model. A stepwise Cox's proportional hazards model was used to simultaneously account for all potential prognostic factors over time. Hazard ratios and two-sided 95% confidence interval (95% CI) were estimated.

## 3. Results

### 3.1. Patients

All tumours were classified according to the contemporary WHO's classification of salivary gland tumours (Barnes et al., 2005). The histological subtype of tumour samples is presented in Table 2, together with prognostic classification and pTN stage.

Among patients with salivary gland cancer, there were 39 men and 31 women aged 61.6 ± 13.9 and 62.0 ± 12.5, respectively. Among control samples, there were 6 women and 4 men, aged 45.0 ± 15.1 and 55.5 ± 9.3, respectively.

The tumours were divided into two groups: (i) favourable clinical prognosis and (ii) poor clinical prognosis. In a group with favourable clinical prognosis there were 38 patients including histological subtypes of salivary gland tumours with low malignant potential: acinic cell carcinomas (ACCCs), mucoepidermoid carcinoma (MEC) grades I and II, epithelial myoepithelial carcinomas (EMCs), and carcinoma ex pleomorphic adenoma (Ca ex PA ) “in situ” (Ca ex PA = 4 cases). In a group with poor clinical prognosis there were 32 patients including histological subtypes of salivary gland tumours with high malignant potential: MEC grade III, adenoid cystic carcinomas (ACCs), adenocarcinoma NOS (ACNOS), poorly differentiated carcinoma, salivary duct carcinomas (SDCs), carcinosarcoma, adenosquamous carcinoma, invasive Ca ex PA (Ca ex PA = 5 cases), and small cell carcinoma (SCC).

### 3.2. Immunohistochemical Expression of EGFR

**Figure 1:**
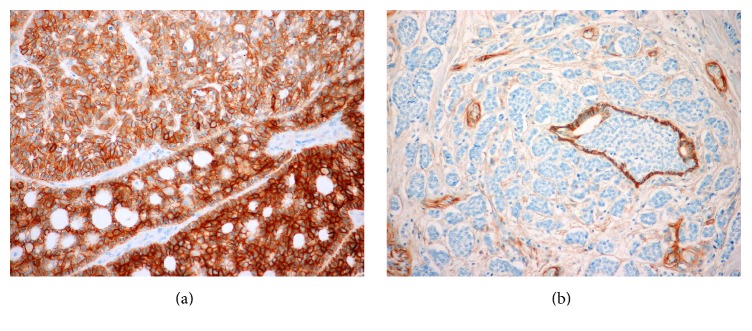
EGFR expression in adenoid cystic carcinoma (ACC) in two different patients. (a) Intense positivity in all cells of parotid gland ACC (EGFR, ×20); (b) EGFR negative ACC cells surround and infiltrate the EGFR positive ductal structure of parotid gland (EGFR, ×20).

Immunohistochemical (positive/negative) expression of EGFR among different histological subtypes is presented in Table 3. Expression of EGFR was found in 14 (37%) samples among favourable clinical prognosis and in 13 (40%) samples among poor clinical prognosis. Examples of cases with positive and negative expression are represented in Figure 1.

### 3.3. Mutation Status and Copy Number Variation of EGFR

In salivary gland tumours we have not found any nucleotide changes being responsible for activation of EGFR and its elevated expression pattern.

According to results of copy number variation (CNV) analysis using qPCR we found in the majority of samples polysomy of chromosome 7. There were similar results between groups with favourable and poor clinical prognosis. There were no sample with disomy, 28 samples (80%) with polysomy and 5 samples (14%) with amplification of EGFR in a group with poor clinical prognosis. There were 1 sample with disomy (3%), 25 samples (71%) with polysomy, and 8 samples (23%) with amplification of EGFR in a group with favourable clinical prognosis. There was no statistically significant change in proportion of samples with disomy, polysomy, or amplification between the histological subtypes of poor and favourable clinical prognosis.

However, in three samples CNV analysis using qPCR failed to give the reliable results (1 ACC, 1 SDC, and 1 MEC). FISH analysis of these three samples gave the following result; ACC showed 80% of disomic cells, 15% of cells with high polysomy, and 5% with amplification; SDC showed 56% of disomic cells, 24% of cells with low polysomy, 16% of cells with high polysomy, and 4% with amplification; MEC showed 80% of disomic cells, 8% of cells with low polysomy, 4% of cells with high polysomy, and 8% with amplification. Therefore, all three samples were treated as disomic.

FISH analyses gave similar results as CNV using qPCR. In 3 samples (15%) we observed positive result, with amplification in one case and combined amplification and polysomy in two cases. All other samples were negative, with majority showing low polysomy and a small proportion with disomy.

### 3.4. microRNA Expression Analysis

**Figure 2:**
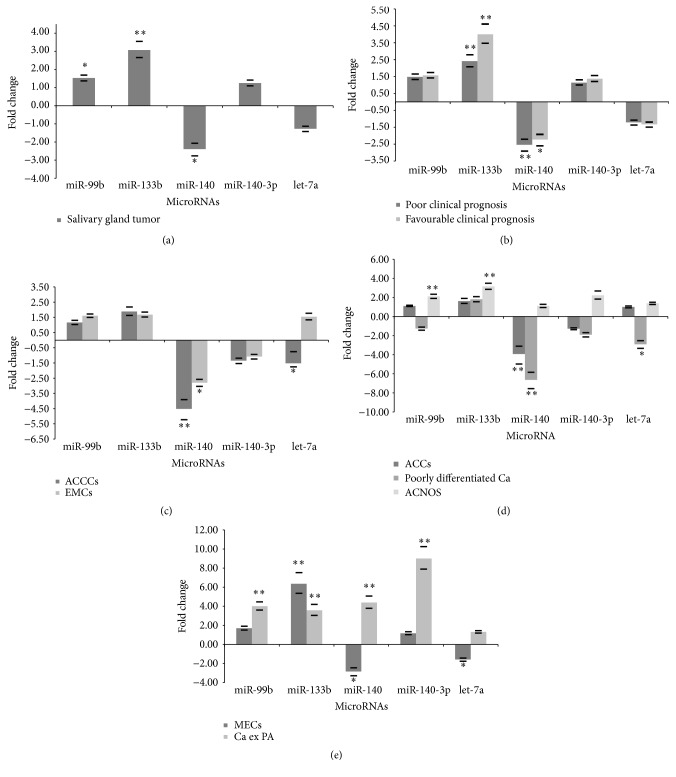
Expression of selected miRNA in salivary gland tumours. Fold changes of miRNAs (a) in salivary gland tumour compared to normal salivary gland; (b) in favourable and poor clinical prognosis compared to normal salivary gland; (c) in ACCC, and EMC compared to normal salivary gland; (d) in ACCs, poorly differentiated Ca, and ACNOS compared to normal salivary gland; (e) in MEC, and Ca ex PA compared to normal salivary gland. Significant expression is marked with asterisk (^*∗∗*^*p* < 0.01, ^*∗*^*p* < 0.05); bars represent the standard deviation.

Analysis of expression of selected miRNA showed that among all salivary gland tumours* miR-140* was downregulated and* miR-133b* and* miR-99b* were upregulated when compared to normal salivary glands (Figure 2(a)). When group with favourable clinical prognosis was compared to normal salivary glands the expression pattern of* miR-133b* was upregulated and expression of* miR-140* and* let-7a* was downregulated (Figure 2(b)). Comparison of favourable versus poor clinical prognosis group yields no statistically significant difference in expression of miRNAs.

Further analysis of most common entities of salivary gland tumours revealed that* miR-140* and* let-7a* are downregulated in ACCCs. Although the number of samples was small we also observed* miR-140* downregulation in EMCs (Figure 2(c)).

When group with poor clinical prognosis was compared to group of normal salivary gland, there were two differentially expressed miRNAs,* miR-133b* and* miR-99b*, which were both upregulated (Figure 2(b)). Further subdivision according to the histological subtypes of tumours revealed downregulation of* miR-140* in ACCs. Although the number of samples was small we also observed* miR-99b* and* miR-133b* upregulation in ACNOS as well as* miR-140* and* let-7a* downregulation in poorly differentiated carcinoma (Figure 2(d)).

Although some of the samples of MEC and Ca ex PA were included in group with favourable and some in group with poor clinical prognosis, we observed* miR-140* and* let-7a* downregulation and* miR-133b* upregulation in MEC, and upregulation of* miR-140*,* miR-140-3p*,* miR-99b*, and* miR-133b* in Ca ex PA (Figure 2(e)).

All the values of fold changes are provided in supplementary Table S1 (in Supplementary Material available online at https://doi.org/10.1155/2017/9150402).

### 3.5. Correlation of EGFR between Immunostaining, Amplification Status, Expression Analyses of miRNAs, and Clinical and Pathohistological Data

First, we were able to confirm very weak but statistically significant association between CNV of EGFR and IHC expression of EGFR. We also observed weak positive association between expression of* let-7a*,* miR-133b*, and* miR-140* and reactivity of EGFR, as well as between CNV of* EGFR *and almost all investigated miRNAs. The strongest correlations were observed between the expression patterns of* let-7a* and expressions of* miR-140* and* miR-140-3p*. All these statistically significant associations and correlations are summarized in Table 4.

Unsurprisingly, we confirmed a moderate positive association between tumour size/stage and the number of metastases (*r*_*s*_ = 0.54, *p* < 0.01). We have further observed increasing expression of all miRNAs except* miR-140* with tumour size/T stage (from T1 to T3); however, the only statistically different expression was observed for* miR-133b* between T1 and T2 (*p* = 0.03) and accordingly* miR-133b* showed weak positive association with tumour size (*r*_*s*_ = 0.23, *p* = 0.05). Distribution of EGFR reactivity and polysomy/amplification was similar between different tumour sizes.

We have also observed increasing expression of* miR-133b* with the presence of metastases (from N0 to N1 and N2); however, the difference in expression did not reach the statistical significance. Distribution of EGFR reactivity and polysomy/amplification was similar, independent of the presence or absence of metastases.

We did not observe any statistically significant change in expression between patients that survived and those that died due to the presence of neoplasm. More interestingly, we did observe the statistically significant higher percentage of EGFR reactive samples (58%) among patients that died due to the presence of salivary glade tumours compared to those that were still alive (30%) at the time of the time of analysis (*p* = 0.05).

### 3.6. Tumour and Patient Characteristics Associated with Overall Survival

**Figure 3:**
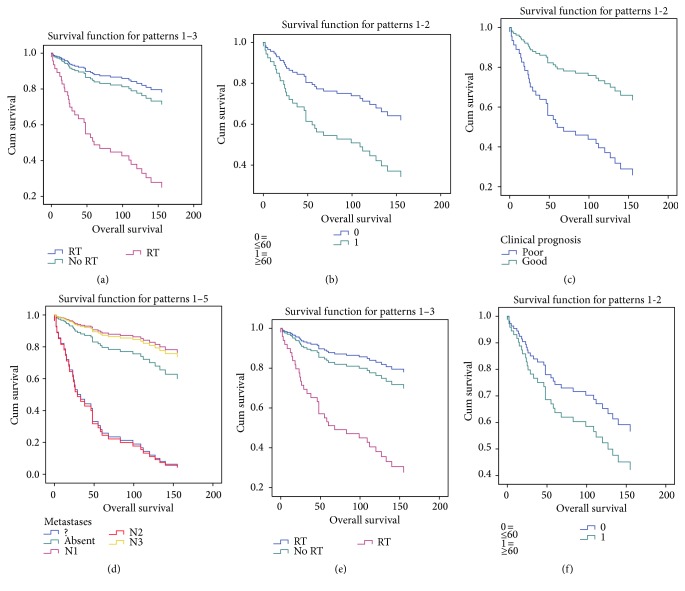
Overall survival of patients based on Cox proportional hazards regression model. Univariate model with impact of (a) radiotherapy (blue line is without data on RT), (b) age (>60 y at the time of diagnosis), (c) poor clinical prognosis, and (d) lymph node infiltration on overall survival. Multivariate model with impact of (e) radiotherapy and (f) age (>60 y at the time of diagnosis). RT, radiotherapy; ≥, more than 60 years; ≤ less than 60 years; N1, N2, and N3, nodal status of TNM tumour classification; ?, no data of lymph node metastases.

The follow-up data was taken on September 25, 2015. Most of the patients have been followed for more than 10 years, and for all patients the follow-up period is more than 5 years. Results on univariate and multivariate analyses are summarized in Table 5. In the univariate model, radiotherapy shows the strongest association with overall survival, followed by age (>60), poor clinical prognosis, and lymph node infiltration. Results are summarized in Figures 3(a)–3(d) and Table 5. In the multivariate analysis, the overall survival is associated with radiotherapy and age (over 60 years at the time of diagnosis). Results are summarized in Figures 3(e) and 3(f) and Table 5. The measured molecular characteristics do not seem to have an effect on the overall survival of the patients.

## 4. Discussion

One of the aims of our study was to investigate expression of EGFR in different subtypes of salivary gland tumours. In 27 of 70 salivary gland tumours we found positive reactivity (3+ IHC staining) of EGFR. Compared to the study performed on ACCs and non-ACCs tumours, the percentage on ACCs was higher in our study (4 of 8) compared to 5 of 20 [3]; however, the overall percentage of EGFR positive reaction was lower (27 of 70) compared to 16 of 39. On a larger cohort of salivary gland tumours [2], 134 of 663 salivary gland carcinomas were found with 3+ EGFR reaction and 80 of 189 salivary gland adenomas with 3+ EGFR reaction [2].

In our study we were able to associate immunohistochemical expression of EGFR with the copy number variation of EGFR (amplification of EGFR or polysomy of chromosome 7), although we did not find any mutation being responsible for activation of EGFR and its elevated expression pattern. Similar to previous research [3], we have observed amplified EGFR in only 2 of 8 ACCs and small proportion among other tumour entities, although half of samples stained 3+ by immunohistochemistry. However, polysomy of chromosome 7 was observed in the majority of the samples. Similar to our results, no samples with amplification and only polysomy were detected on large cohort of salivary gland tumours with only 2 mutations found in 2 samples out of 107 [2].

Distribution of EGFR reactivity and polysomy/amplification was similar between different tumour sizes and independent of metastatic disease. These results are in contrast to the studies performed on larger cohort of patients, where EGFR positivity was associated with tumour size [1] and lymph node metastases [1, 19]. However, one of these two studies, similar to our results, observed that copy number gain predicted worse survival for the patients [1]. Accordingly, our results showed statistically significant higher percentage of EGFR reactive samples (58%) among patients that died due to the presence of salivary gland tumours compared to those that were still alive (30%) at the time of analysis.

In the second part of the study we have analysed expression of five different miRNAs,* miR-99b, miR-133b, miR-140, miR-140-3p,* and* let-7a*, since* miR-99b, miR-140,* and* let-7a* have been shown to be differently expressed in PA of salivary gland [12]. The aim of our study was to analyse what is their expression in other salivary gland tumour subtypes. We further tested whether expression of* miR-133b* is in correlation with the expression of its target, EGFR, which was according to miRTarBase (http://mirtarbase.mbc.nctu.edu.tw, a database of confirmed and validated miRNA targets) validated using Reporter Assay, Western blot, and qPCR.

We observed differences in expression patterns of* miR-133b, miR-140, *and* let-7a* when group with poor clinical prognosis was compared to normal salivary glands. When group with favourable clinical prognosis was compared to group of normal salivary gland there were two differentially expressed miRNAs,* miR-133b* and* miR-99b*. Further analysis of most common entities of salivary gland tumours revealed downregulation of* miR-140* in MECs, ACCCs, EMCs, and ACCs and in poorly differentiated carcinoma;* let-7a* in MECs and ACCCs and in poorly differentiated carcinoma; and* miR-99b* in poorly differentiated carcinoma. We further observed upregulation of miR-133b in MEC and in ACNOS and* miR-99b* in ACNOS. Similar to a previous described study on PAs [12], differentially expressed miRNAs in Ca ex PA appears to be upregulated* miR-140, miR-140-3p, miR-99b,* and additionally* miR-133b*. However, downregulation of* let-7a* was not observed.

In association of miRNAs expression with the immunohistochemical expression of EGFR, we observed positive association between expression of* miR-133b *and* let-7a* and reactivity of EGFR.* Let-7a* has been already implicated in tumourigenesis and similarly as in MECs, ACCCs, and poorly differentiated carcinoma of salivary glands it has been shown to be reduced in lung cancer. Its two targets appear to be RAS and HMGA2, oncogenic proteins in a variety of tumours [12]. It has been recently observed that WIF1 (Wnt inhibitory factor 1) is downregulated in Ca ex PA and that when overexpressed it increases expression of pri-let-7a, a primary transcript of* let-7a* [20].* miR-133b* has been downregulated in many tumours and upregulated only in cervical carcinoma, where it acts as an oncogene to promote tumorigenesis and metastasis. Its targets are MST2, CDC42, and RHOA, which results in activation of AKT and ERK signalling pathways [21]. In research done on non-small-cell lung cancer the expression of* miR-133b* also influenced EGFR signalling pathway [14].

Additionally, we have found positive association between* miR-133b, miR-140, miR-140-3p*, and* let-7a* and CNV of EGFR.* miR-140* is believed to be cartilage specific miRNA; however, it appears to be important in certain subtypes of salivary gland tumours and for differential diagnosis of histological subtypes of salivary gland tumours. Pronounced positivity of* miR-140* in Ca ex PA, which most probably reflects the presence of remaining chondroid matrix, a usual component of PA, could therefore in the differential diagnostic process implicate a malignant transformation of PA in comparison with a tumour arising “de novo,” where we would anticipate negative values of* miR-140*. Using intersection of mRNAs repressed by* miR-140* overexpression and of mRNAs expressed by* miR-140* silencing, 49 genes were identified as potential* miR-140* targets with 22 of these possessing seed sequence [22]. Therefore, which of these is implicated in salivary gland tumour is yet to be identified.

We have also observed increasing expression of all miRNAs except* miR-140* with tumour size (from T1 to T3); however, the only statistically different expression was observed for* miR-133b* between T1 and T2 and accordingly* miR-133b* showed positive correlation with tumour size. There was also an increasing expression of* miR-133b* with the presence of metastases (from N0 to N1 and N2) although the difference in expression did not reach the statistical significance.

Overall survival analysis showed that statistically significant parameters influencing overall survival are of clinical nature. In univariate analysis those factors are age above 60 years at the time of the diagnosis, lymph node metastases, poor clinical prognosis, and radiotherapy. Multivariate analysis showed that radiotherapy and age above 60 years have significant impact on overall survival. These results are similar to those previously published, although they also included the proliferative marker Ki67 [23]. We did not observe any statistically significant change in expression patterns between patients that survived and those that died due to the presence of neoplasm; however, we did observe upregulation of* miR-133b* in patients that died due to other reasons compared to those that were alive at the time of analysis. We were able to confirm the correlation of expression of* miR-133b* with the immunohistochemical expression of its target.

## 5. Conclusions

In summary, we observed expression of EGFR in 37% of tumours with low malignant potential and in 40% of tumours with high malignant potential. There were no mutations in* EGFR*; in the majority of samples (76%) polysomy of chromosome 7 was detected, and amplification was present in 19% of samples. In demonstrated absence of EGFR gene mutations in malignant salivary gland tumours, we can conclude that variability in EGFR expression occurs as a consequence of gene amplification and chromosome 7 polysomy. Although no significant differences were observed among different histologic types, further studies are needed to eliminate EGFR as a possible therapeutic target in malignant salivary gland tumours. Further “negative” results would push us and other researchers toward identification of novel biomarkers and biological targets in salivary gland tumours.

The expression of* miR-99b* and* miR-133b* was upregulated, while* miR-140* expression was downregulated in salivary gland tumours compared to normal salivary glands.* miR-133b* was upregulated in poor and favourable clinical prognosis compared to normal tissues; additionally in the poor clinical prognosis group upregulation was observed in expression of* miR-99b* and in favourable clinical prognosis group the downregulation of* miR-140*. In all histological subtypes,* miR-140* was significantly down-regulated, except in Ca ex PA, where it was significantly up-regulated.* miR-133b* was significantly upregulated in ACNOS, Ca ex Pa, and MEC subtypes;* miR-99b* was also upregulated in ACNOS and Ca ex PA, while* miR-140-3p* expression was only significant in Ca ex PA. Downregulated expression profile of* let-7a* was observed in ACCC, MEC, and poorly differentiated Ca.

We observed positive correlation between EGFR reactivity,* miR-133b*,* miR-140*,* miR-140-3*, and* let-7a* and CNV of EGFR and a positive correlation between* miR-133b* and* let-7a* and reactivity of EGFR.

Univariate overall survival analysis showed that age, lymph node infiltration, radiotherapy, and clinical prognosis have significant effect on survival, while multivariate approach yields age and radiotherapy as significant factors. There was also a statistical difference in expression of* miR-133b* between T1 and T2 tumour size.

Differential expression of various microRNAs in malignant salivary gland tumours, especially upregulation of* miR-140,* appears to be important in the differential diagnosis, pointing to possible malignant transformation of PA.

## Supplementary Material

Table S1: Fold changes of miRNAs expression in salivary gland tumours. Arrow indicates up- or down-regulated expression. Significance is marked with asterisk (^**^p < 0.01, ^*^p < 0.05).

## Figures and Tables

**Table 1:** Sequences of primer pairs used for PCR.

Exon	Primer sequence	Genomics position
18	5′-CATGGTGAGGGCTGAGGTGA-3′	chr7: 55173871–55173890
	5′-GTCCCTGGCACAGGCCTCTGG-3′	chr7: 55174049–55174069
19	5′-ATGTGGCACCATCTCACAATTGCC-3′	chr7: 55174667–55174690
	5′-AAAGGTGGGCCTGAGGTTCAGA-3′	chr7: 55174856–55174877
20a	5′-AAGCCACACTGACGTGCCTCT-3′	chr7: 55181260–55181280
	5′-ACCGTGCAGCTCATCACGC-3′	Chr7: 55181362–55181380
20b	5′-CCTCCACCGTGCACCTCATC-3′	chr7: 55181357–55181377
	5′-CCCGTATCTCCCTTCCCTGA-3′	chr7: 55181483–55181502
21	5′-GGATGCAGAGCTTCTTCCCATGAT-3′	chr7: 55191659–55191682
	5′-AAATGCTGGCTGACCTAAAGCCAC-3′	chr7: 55191883–55191906

**Table 2:** Tumour characteristics at diagnosis.

Characteristics (*n* = available data)	Number of patients (%)
Histological subtype (*n* = 70)	
Acinic cell carcinoma (ACCC)	16 (22.9)
Mucoepidermoid carcinoma (MEC)	16 (22.9)
Carcinoma ex pleomorphic adenoma (Ca ex PA)	9 (12.9)
Adenoid cystic carcinoma (ACC)	8 (11.4)
Poorly differentiated carcinoma	7 (10.0)
Epithelial myoepithelial carcinoma (EMC)	5 (7.1)
Adenocarcinoma NOS (ACNOS)	4 (5.7)
Salivary duct carcinoma (SDC)	2 (2.9)
Small cell carcinoma (SCC)	1 (1.4)
Carcinosarcoma	1 (1.4)
Adenosquamous carcinoma	1 (1.4)
Classification (*n* = 70)	
Favourable clinical prognosis	35 (50.0)
Poor clinical prognosis	35 (50.0)
Tumour size/T stage (*n* = 67)	
Tis	4 (6.0)
T1	15 (22.4)
T2	18 (26.9)
T3	14 (20.9)
T4	16 (23.9)
Lymph node status/N stage (*n* = 64)	
N0	45 (70.3)
N1	5 (7.8)
N2	14 (21.9)
Surgical resection (*n* = 63)	59/63 (93.7%)
Postoperative radiotherapy (*n* = 63)	39/63 (61.9%)

**Table 3:** Number of samples with positive and negative EGFR expression pattern and copy number variation of EGFR among histological subtypes of salivary tumours.

Histological subtype	EGFR, 3+ (%)	CNV EGFR
		Amplification, *n* (%)
		Polysomy, *n* (%)
		Disomy, *n* (%)
MECs (*n* = 16)	8 (50)	4 (25.0)
		11 (68.8)
		1 (6.3)

ACCCs (*n* = 16)	2 (11.1)	3 (18.8)
		12 (75)
		1 (6.3)

Ca ex PA (*n* = 9)	6 (66.7)	1 (11.1)
		8 (88.9)
		0

ACCs (*n* = 8)	4 (50)	2 (25.0)
		5 (62.5)
		1 (12.5)

Poorly differentiated carcinoma (*n* = 7)	3 (42.9)	2 (28.6)
		5 (71.4)
		0

EMCs (*n* = 5)	4 (80.0)	0
		5 (100)
		0

ACNOS (*n* = 4)	0	1 (25.0)
		3 (75.0)
		0

SDCs (*n* = 2)	0	0
		1 (50)
		1 (50)

SCC (*n* = 1)	0	0
		1 (100)
		0

Carcinosarcoma (*n* = 1)	0	0
		1 (100)
		0

Adenosquamous carcinoma (*n* = 1)	0	0
		1 (100)
		0

Total	27/70 (38.6)	13/70 (18.6)
		53/70 (75.7)
		4/70 (5.7)

**Table 4:** Correlations/associations between CNV of EGFR and IHC expression of EGFR and microRNA expression. Significant correlations/associations are marked with asterisk (^*∗∗*^*p* < 0.01, ^*∗*^*p* < 0.05).

	CNV of EGFR	IHC of EGFR	ΔCt *miR-99b*	ΔCt *miR-133b*	ΔCt *miR-140*	ΔCt *miR-140-3p*	ΔCt *let-7a*
CNV of EGFR							
IHC of EGFR	0.13^*∗∗*^						
ΔCt (*miR-99b*)	−0.07^*∗∗*^						
ΔCt (*miR-133b*)	0.11^*∗∗*^	0.12^*∗∗*^	0.42^*∗*^				
ΔCt (*miR-140*)	0.25^*∗*^	0.03^*∗*^		0.24^*∗∗*^			
ΔCt (*miR-140-3p*)	0.27^*∗*^			0.28^*∗*^			
ΔCt (*let-7a*)	0.21^*∗∗*^	0.31^*∗∗*^		−0.08^*∗*^	0.50^*∗*^	0.48^*∗*^	

**Table 5:** Association between tumour characteristics and overall survival. Univariate analysis (Cox proportional hazards regression model) is shown in the left panels and multivariate analysis (Cox regression with stepwise backwards selection) in the right panels. Statistically significant *p* values in the univariate analysis and in the last step of the multivariate analysis are shown in bold. HR, hazard ratio; CI, confidence interval.

Characteristics (*n* = available data)	Univariate model			Multivariate model		
	HR	95% CI	*p* value	HR	95% CI	*p* value
Male (*n* = 70)	0.57	0.28–1.16	0.12			
Age > 60 years (*n* = 70)	**2.88**	**1.29**–**6.43**	**0.01**	**2.22**	**0.97**–**5.08**	**0.05**
Tumour size ≥ 2 cm (*n* = 63)	1.56	0.67–3.63	0.36			
Lymph node infiltration (*n* = 63)	**1.84**	**1.25**–**2.73**	**0.001**			
Radiotherapy (*n* = 61)	**3.60**	**1.48**–**8.76**	**0.005**	**2.91**	**1.17**–**7.25**	**0.02**
Poor clinical prognosis (*n* = 70)	**2.01**	**1.00**–**4.01**	**0.049**			
ICH EGFR (*n* = 70)	1.00	0.99–1.00	0.10			
CNV EGFR (*n* = 70)	0.93	0.80–1.08	0.35			
ΔC_t_ *miR-99b*	1.05	0.80–1.38	0.71			
ΔC_t_ *miR-133b*	0.93	0.82–1.06	0.27			
ΔC_t_ *miR-140*	1.04	0.87–1.24	0.70			
ΔC_t_ *miR-140-3p*	1.01	0.84–1.22	0.89			
ΔC_t_ *let-7a*	1.01	0.76–1.34	0.93			
