# Supplementary material for: Expression, Mutation, and Amplification Status of EGFR and Its Correlation with Five miRNAs in Salivary Gland Tumours
Source: Biomed Res Int. 2017 Mar 9;2017:9150402. doi: 10.1155/2017/9150402 (PMC5362712; doi:10.1155/2017/9150402)
Supplement: Supplementary file 1 — Table S1: Fold changes of miRNAs expression in salivary gland tumours. Arrow indicates up- or down-regulated expression. Significance is marked with asterisk (**p < 0.01, *p < 0.05). [file 9150402.f1.pdf]

Supplementary material

Table S1: Fold changes of miRNAs expression in salivary gland tumours. Arrow indicates up- or down-regulated expression. Significance is marked with asterisk (** p < 0.01, * p < 0.05).

	miR-99b	miR-133b	miR-140	miR-140-3p	Let-7a
Tumour vs. normal	1.53*↑	3.07**↑	2.39*↓		
Poor clinical prognosis vs. normal	1.73*↑	2.52**↑			
Favourable clinical prognosis vs. normal		3.91**↑	2.94**↓		1.50*↓
ACCC vs. normal			4.52**↓		1.52*↓
EMC vs. normal			2.80*↓		
ACC vs. normal			3.93**↓		
ACNOS vs. normal	2.11**↑	3.16**↑			
Poorly differentiated Ca vs. normal			6.64**↓		2.90*↓
MEC vs. normal		6.35**↑	2.83*↓		1.58*↓
Ca ex PA vs. normal	4.01**↑	3.57**↑	4.39**↑	9.00**↑	
